# Adamantinoma-Like Ewing Sarcoma of the Parotid Gland: Report of Two Cases and Review of Literature

**DOI:** 10.7759/cureus.11870

**Published:** 2020-12-03

**Authors:** Hala Alnuaim, Malak Alzahrani, Samirah Ghandurah, Mohammad Dababo

**Affiliations:** 1 Pathology and Laboratory Medicine, King Abdulaziz Medical City, Riyadh, SAU; 2 Anatomical Pathology, King Saud University, Riyadh, SAU; 3 Anatomic Pathology, King Faisal Specialist Hospital & Research Centre, Riyadh, SAU

**Keywords:** parotid gland, salivary gland, ewing, sarcoma, adamantinoma

## Abstract

Adamantinoma-like Ewing sarcoma (ALES) is a rare variant of Ewing sarcoma. It demonstrates heterogeneous morphologic pattern and complex immunophenotypic profile, with a peculiar combination of epithelial and neuroendocrine differentiation. ALES is rarely reported in the head and neck areas, including the parotid salivary gland. Till now, only 10 cases of ALES have been reported in the salivary glands. Herein, we report two cases of ALES involving the parotid gland, adding some valuable insight to the recently reported cases at this site.

## Introduction

Ewing sarcoma (ES) is a small round cell tumor typically reported in bone and soft tissues at a young age group. In addition to their morphological and immunophenotype features, these tumors are defined by the demonstration of EWSR1 gene rearrangement. This occurs due to a translocation between EWSR1 and a gene of the ETS family of transcription factors, of which FLI1 constitutes about 85% of fusion partners [1]. Fluorescence in-situ hybridization testing (FISH) detects this rearrangement by using the break apart probe in the EWSR1 gene, thereby, indirectly indicating the translocation. The reverse transcriptase polymerase chain reaction (RT-PCR) is the most effective method for detecting the exact fusion partner of the EWSR1 gene [1,2]. Approximately, 5% of ES develops in the head and neck region, and up to 20% might show some epithelial differentiation [2,3]. Adamantinoma-like Ewing sarcoma (ALES) was first described by A. Folpe in three cases of bone/soft tissues exhibiting a complex histopathological and immunophenotypical epithelial differentiation [4]. Rare cases of ALES were reported in the head and neck regions, including salivary glands (eight tumors arose in parotid glands and two in submandibular glands), sinonasal tract, orbit and thyroid gland [2,5,6]. Morphologically, these tumors show nested, solid and lobular patterns of monotonous basaloid cell proliferation with desmoplastic fibrous stroma, brisk mitotic activity and foci of necrosis. CD99 along with positive staining for cytokeratin, mainly CK5/6, and strong diffuse positivity for p63 and p40 are evident in tumor cells. FISH testing for EWSR1 gene rearrangement was found to be positive in all cases [2].

## Case presentation

First case

**Figure 1:**
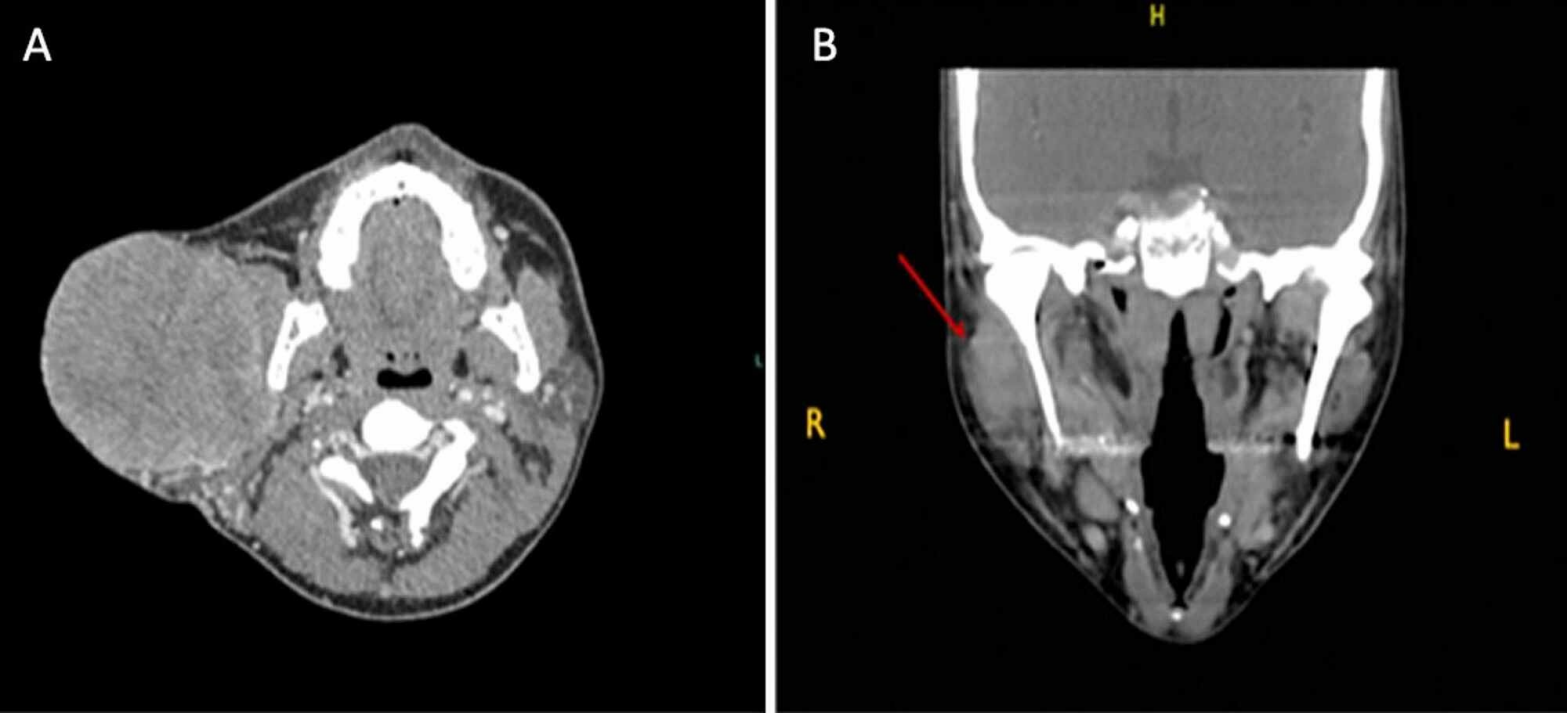
CT head and neck with contrast (A) First case: CT head and neck with contrast showing right parotid gland mass measuring 8.5 × 8.4 × 8.3 cm with central necrosis and mass effect on sternocleidomastoid muscle. (B) Second case: neck CT scan showing poorly demarcated mass (arrow) occupying superficial lobe of right parotid gland.

**Figure 2:**
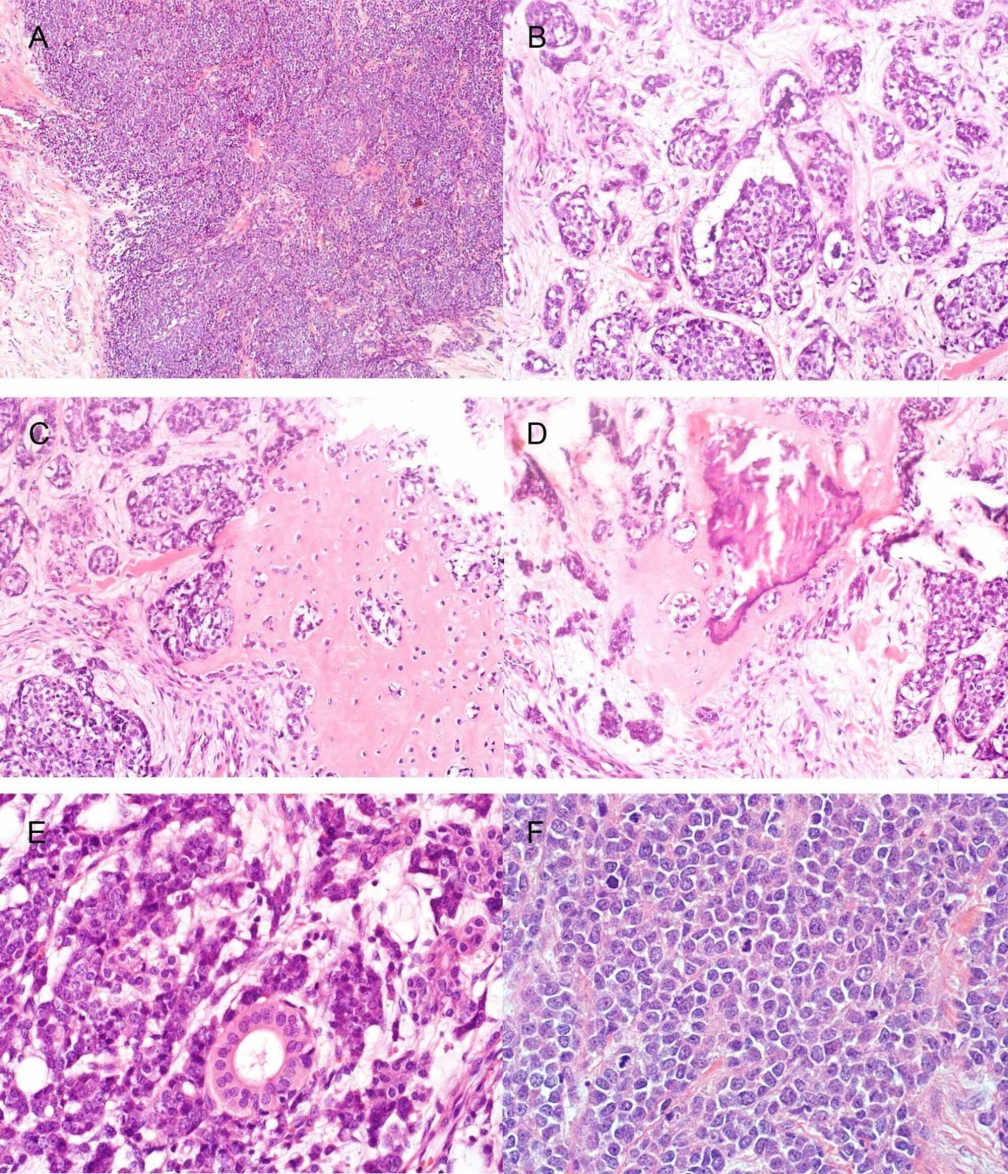
H&E of the first case (A) Tumor cells forming solid sheets. (B) Tumor cells arranged in nests. (C) Chondroid differentiation. (D) Chondro-osseous differentiation. (E) Normal duct entrapped within the tumor. (F) Mitosis. H&E, hematoxylin and eosin.

**Figure 3:**
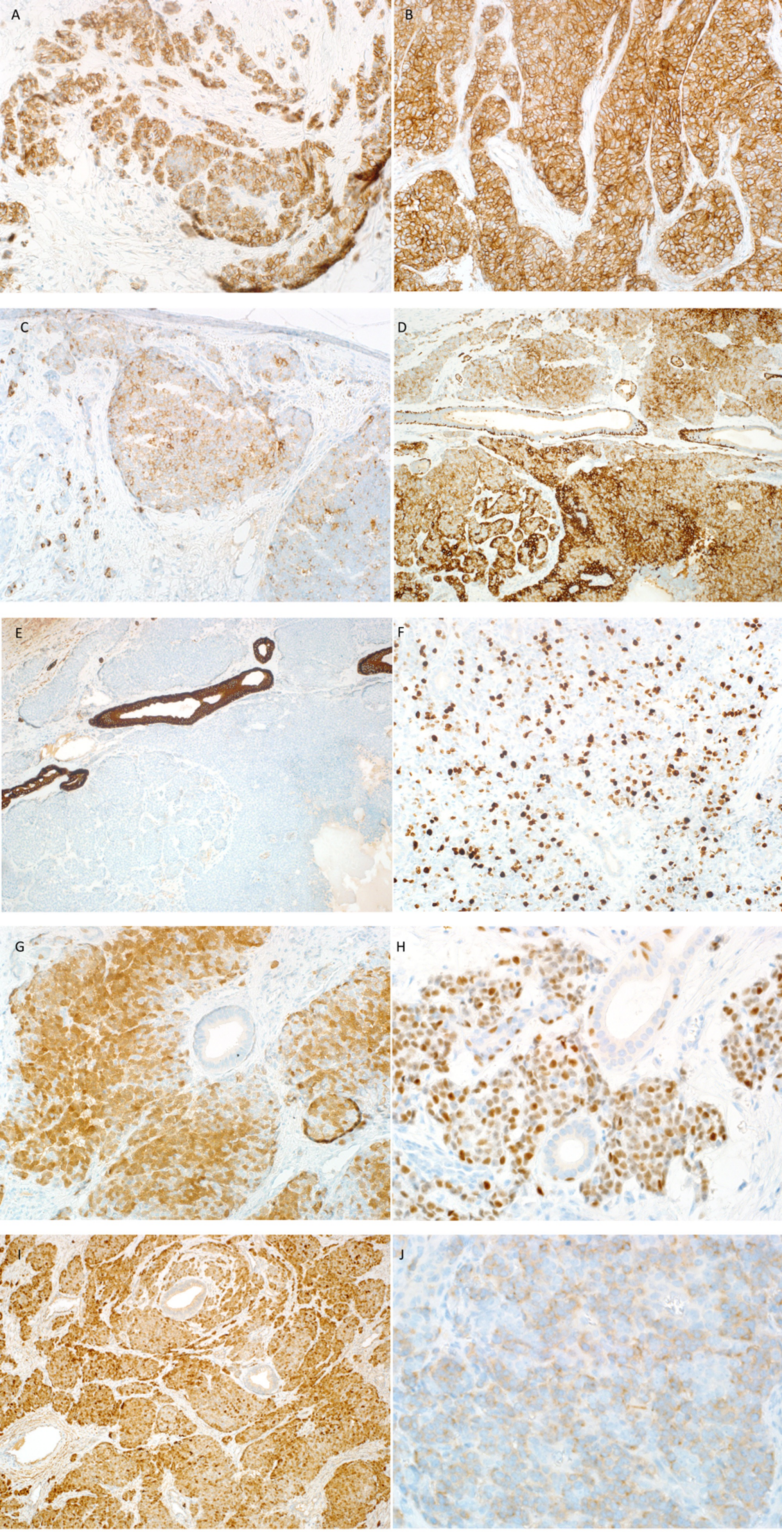
IHC stains of the first case (A) CD56: focally positive. (B) CD99: strong and diffuse membranous positivity in tumor cells. (C) Chromogranin: focally stains positive. (D) CK5/6: highlights myoepithelium of normal duct and stains tumor cells. (E) CK7: negative staining in tumor cells and positive in normal salivary duct. (F) Ki67: positive in more than 20%. (G) P16: nuclear and cytoplasmic positive staining in more than 75% of tumor cells. (H) P63: diffuse positive staining in tumor cells surrounding normal ducts. Some positive staining is also seen in myoepithelial cells. (I) PGP9.5: positive. (J) Synaptophysin: weakly positive. IHC, immunohistochemistry.

A 29-year-old male presented with a year history of right cheek mass that was gradually increasing in size. A superficial parotidectomy was initially done in another hospital, and subsequently, was diagnosed as adenocarcinoma. He was presented to our institution with residual disease. Computed tomography (CT) with contrast revealed an ill-defined mass measuring 8.8 cm in greatest dimension in the right parotid gland with central necrosis. It was invading the overlying skin and bulging into the right neck space (Figure 1A). CT scans showed no radiological evidence of metastasis. Reviewing of his prior pathology showed a neoplasm predominantly comprised of small round blue cells arranged in solid nests and separated by fibrous stroma (Figure 2A, B). Tumor nests were composed of monomorphic cells with indistinct cytoplasm and nuclei with finely granular chromatin, occasional prominent nucleoli and high mitotic rate (Figure 2F). Focal area of chondro-osseous differentiation was identified (Figure 2C, D). Immunohistochemical staining showed positivity of tumor cells for P16, CK5/6, CK8/18, P63, CD99 (strong and diffuse membranous staining), PGP9.5, and focally for CK20, chromogranin and CD56. Synaptophysin was weakly positive and Ki67 showed >20% proliferation index (Figure 3). However, tumor cells were negative for CDX2, TTF1, GFAP, CK7 and desmin (Table 1). Based on the morphological and immunohistochemical features, FISH for EWSR1 gene rearrangement was performed, and positive results were obtained. EWSR1-FLI1 fusion transcript gene rearrangement was detected by RT-PCR confirming the diagnosis of ALES. Accordingly, the patient was treated with five cycles of chemotherapy. Post-treatment, the patient showed partial response in terms of tumor size. Subsequently, right radical parotidectomy with neck dissection was performed. Gross evaluation revealed a poorly demarcated nodular mass with white cut surface. Microscopic examination demonstrated the same histological features as diagnosed previously for ALES. The resection showed positive resection margins, angioinvasion and perineural invasion. Postoperatively, the patient received adjuvant chemotherapy and radiation therapy. Twenty-two months after the radical resection, the patient presented with local recurrence, multiple pelvic-abdominal deposits and spinal bone metastatic lesions. Patient was referred to palliative care for continuing symptomatic management.

 

**Table 1:** Summary of immunohistochemical results of both cases

Immunohistochemical stains	Clone	First case	Second case
CD56	123C3	+ (focal)	-
CD99	O13	+ (diffuse and strong)	+
Chromogranin	LK2H10)	+ (focal)	-
CK5/6	D5/1684	+	+
CK7	SP52	-	-
Ki67	30-9	+ (>20% of tumor cells)	Not done
P16	CINTecP16	+ (>75%)	+ (focal)
P63	4A4	+ (Diffuse)	+ (diffuse)
PGP 9.5	10-A1	+	Not done
Synaptophysin	SP11	+ (weak)	+ (patchy)

Second case

**Figure 4:**
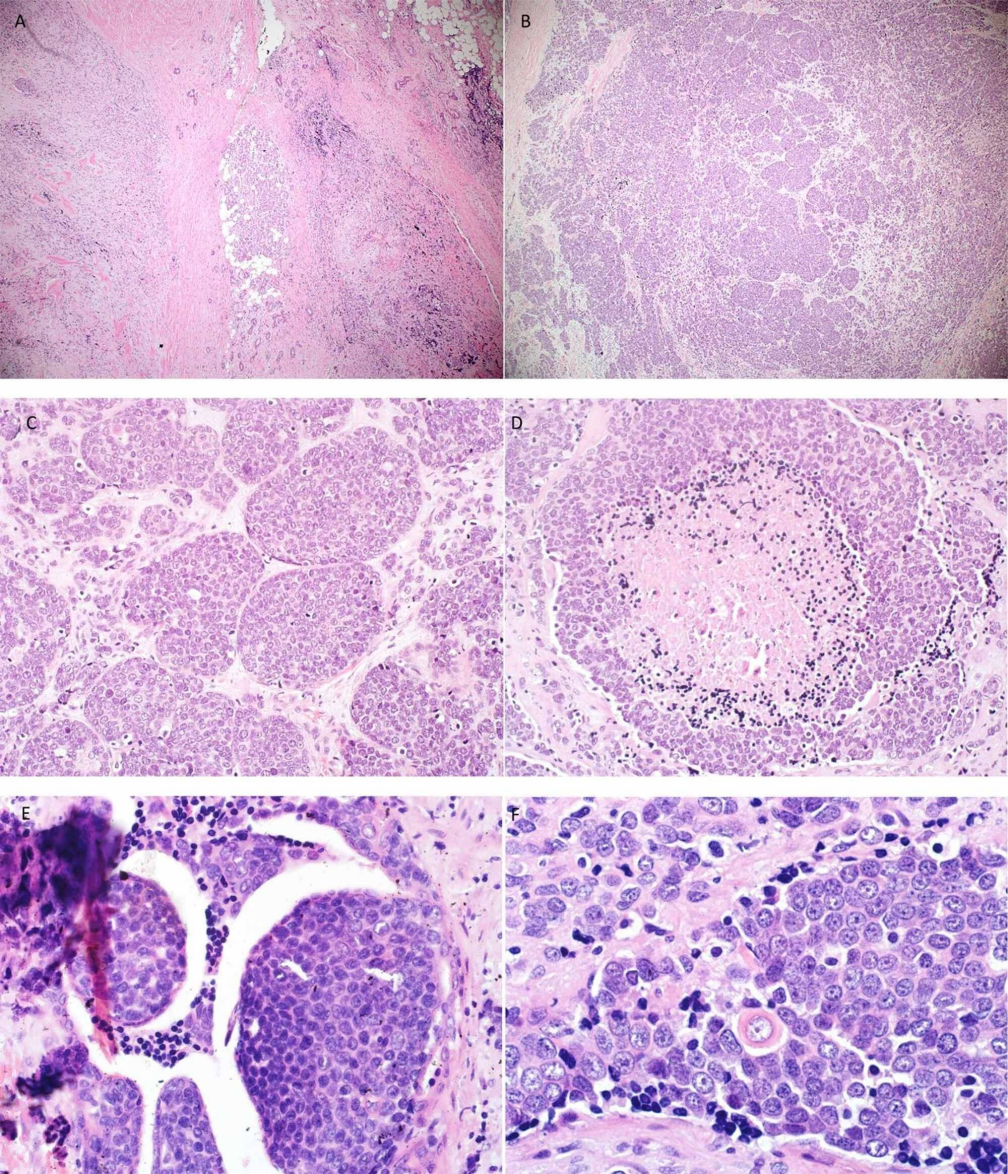
H&E of the second case (A) Tumor cells infiltrating salivary gland parenchyma. (B, C) Tumor cells arranged in nests and lobules. (D) Tumor necrosis. (E) Tumor cells extending into benign duct. (F) Very rare cell with squamous differentiation. H&E, hematoxylin and eosin.

**Figure 5:**
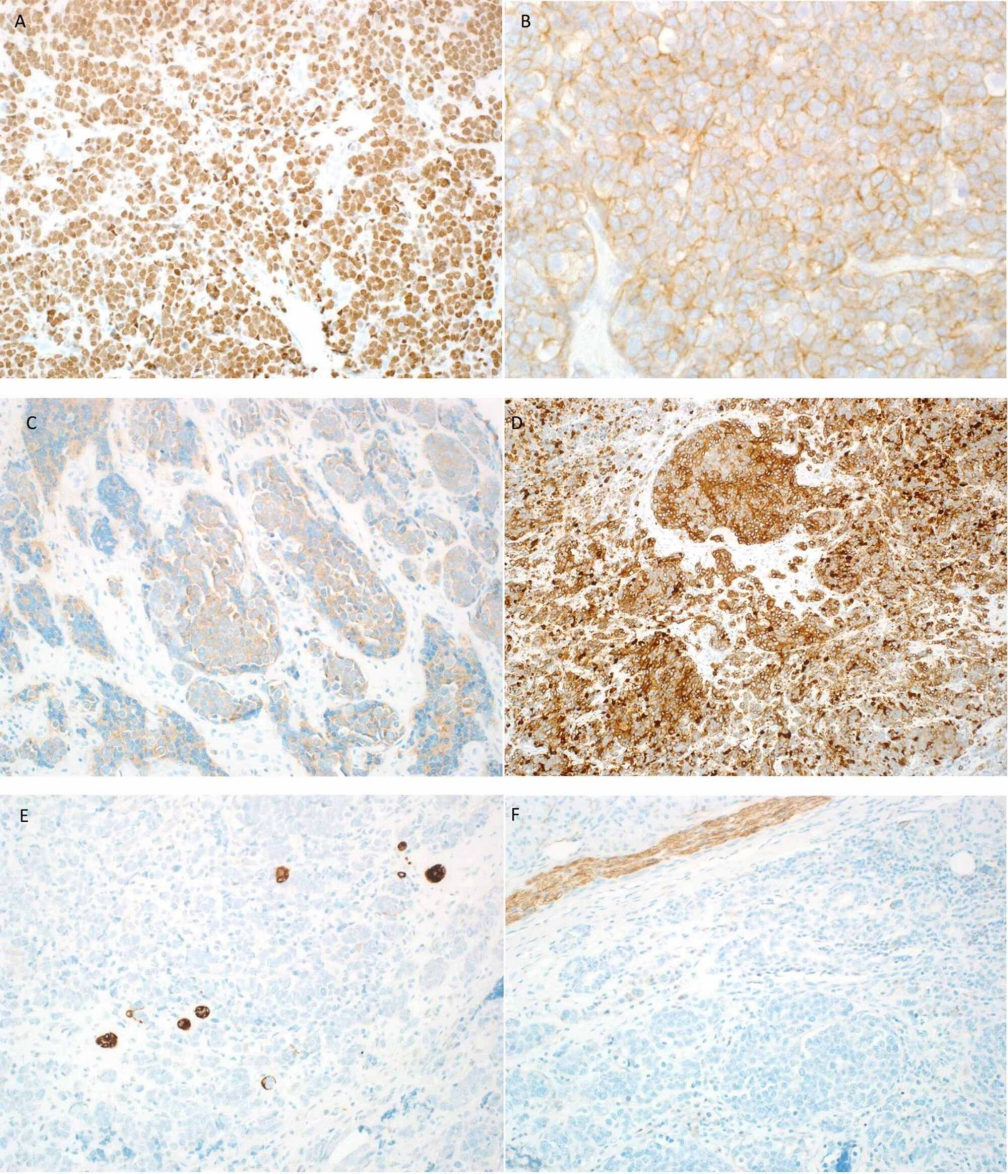
IHC stains of the second case (A) P63: diffuse membranous positivity. (B) CD99: diffuse membranous positivity. (C) Synaptophysin: focally and weakly positive in tumor cells. (D) CK5/6: diffusely positive in tumor cells. (E) CK7: negative in tumor cells and positive in the entrapped ducts. (F) CD56: negative in tumor cells and highlights nerve as internal control. IHC, immunohistochemistry.

**Figure 6:**
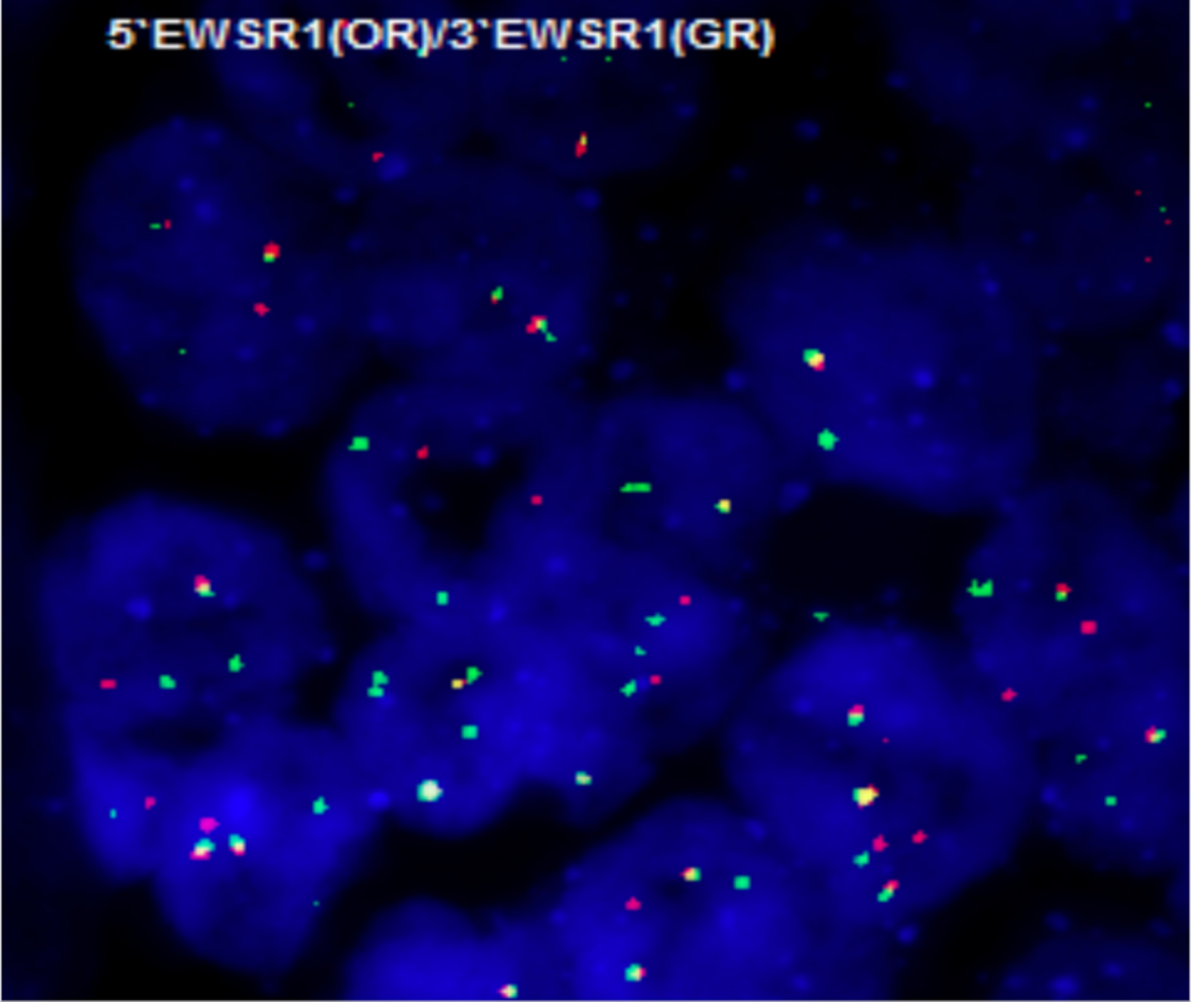
FISH break apart probe showing EWSR1 rearrangement FISH, fluorescence in-situ hybridization.

A 46-year-old female presented with a history of swelling behind the right ear over the parotid area for two years. The case was a referral from outside hospital to our institution. She had a history of multifocal papillary thyroid microcarcinoma seven years before, for which a total thyroidectomy was done, and radioactive iodine was given. Radiologically, a solid mass measuring 2.0 cm in greatest dimension was identified, occupying the superficial lobe of the right parotid gland (Figure 1B). A right total parotidectomy with facial nerve and neck dissection were subsequently performed, and the specimen was submitted for pathologic evaluation. The referring hospital had diagnosed the patient with poorly differentiated carcinoma with neuroendocrine features. Microscopic examination of the outside slides, submitted to our institution for revision of diagnosis, demonstrated a nested basaloid proliferation infiltrating the gland parenchyma with significant fibrosis and desmoplasia (Figure 4A, B). Tumor surrounded and extended into some salivary ducts but did not appear to originate from these ducts (Figure 4E). Tumor cells were monomorphic with fine chromatin and small prominent nucleoli. Some areas showed stippled salt and pepper chromatin reminiscent of neuroendocrine type of chromatin (Figure 4C). Occasional areas of focal necrosis, lymphovascular invasion and mitosis were also found (Figure 4D). Focal areas of pseudorosettes were evident. Occasionally, scattered cells with abundant eosinophilic cytoplasm, representing squamous differentiation, were seen (Figure 4F). A specimen submitted as fascial nerve was also found to be involved in the tumor. Immunohistochemically, tumor cells showed diffuse positivity for p63. However, CK5/6 and CD99 showed strong and diffuse membranous staining by the majority of tumor cells. Staining with synaptophysin showed patchy positivity (Figure 5). Vimentin, chromogranin, TTF1, CD56, HER2, CD117, and CK7 were negative. (Table 1). FISH for the EWSR1 gene rearrangement was positive in 100% of the nuclei (Figure 6). Patient received concurrent chemo-radiotherapy cycles and travelled abroad to continue her treatment.

## Discussion

ALES is a rare tumor with predilection to the head and neck areas [6]. This diagnosis is challenging due to its complex epithelial differentiation consisting of basaloid nests of small monomorphic cells with diffuse keratin and p40 immunohistochemical expression. In addition, this tumor can also show stippled chromatin staining pattern with expression of neuroendocrine markers, such as synaptophysin and CD56. The latter feature may create a diagnostic challenge with neuroendocrine carcinoma. The other possible considerations include NUT midline carcinoma characterized by proliferation of small-sized primitive cells with areas of abrupt keratinization. However, NUT carcinoma is typically negative for CD99 and has a consistent nuclear localization of NUT immunohistochemical stain. Small cell neuroendocrine carcinoma and basal cell adenocarcinoma may also be found in tumor site. Although small cell neuroendocrine carcinoma stains positive for synaptophysin, the CD99 and p63 stains are negative. Basal cell adenocarcinoma of salivary glands is typically of low grade and is nonreactive to CD99 [5].

The differential diagnosis of adamantinoma-like EFT is not limited to epithelial neoplasms. Small round blue cell tumors should also be considered in the differential diagnosis. Desmoplastic small round cell tumors are rarely found in the head and neck regions and usually express CD99, cytokeratin, desmin and WT-1. However, they are negative for p63 and p40 expressions [7,8]. These tumors also harbor translocations involving the EWSR1 gene with WT1 as a fusion partner, which can be detected by RT-PCR. Poorly differentiated synovial sarcoma is another diagnostic consideration that usually exhibits epithelial differentiation in morphology and immunohistochemical examination. It should be noted that synovial sarcoma can also be positive for CD99 but is negative for p63, p40 and neuroendocrine markers. It is characterized by translocations involving the SYT gene rather than EWSR1. Also, lymphoid neoplasm should be considered as one of the round blue cell tumors. In the diagnosis of lymphoma, one should be attentive to CD99 positivity in lymphoblastic lymphoma.

The unique nuclear monotony of ALES is reported to be an important feature in the correct reorganization and differentiation from the more commonly encountered higher grade tumors in the head and neck area [6]. Once the diagnosis of ALES is executed, diffuse membranous IHC staining for CD99 further supports the diagnosis. At the molecular level, it is characterized by EWSR1 - FLI1 translocation. In this article, we reported two cases of ALES. The first case was initially diagnosed as poorly differentiated carcinoma. Microscopically, the tumor was composed of solid nests of monomorphic small round blue cells with a separating dense fibrous stroma. Upon re-investigation, it was found to be CD99 positive in addition to the expression of cytokeratin and neuroendocrine markers (Table 1). A unique finding, in this case, was the presence of a focal area of chondro-osseous differentiation, representing a heterologous element. This specific finding has not been previously described in ALES. Subsequently, the case was sent for RT-PCR to detect the fusion partner, which was consistent with EWSR1-FLI1. The second case has been also referred to our institution with a diagnosis of poorly differentiated neuroendocrine carcinoma. Focal squamous differentiation reported in this patient is not commonly encountered [6]. The largest published case series by Rooper et al showed that this finding has only been described in one out of 10 reported cases [6]. Both of our cases underwent surgical excision, followed by chemoradiotherapy regimen for ES. Most of the reported cases in the literature followed an indolent course with no evidence of disease recurrence during the follow-up period, which ranged from one month to 24 months [6]. However, clinical follow-up of our first patient showed disease progression, initially with localized recurrence and subsequent development of distant bone metastasis. However, our second case was free of disease till three months but later we lost follow-up as the patient traveled abroad to continue her management.

## Conclusions

In conclusion, although ALES is a rare and recently described entity, it carries a peculiar combination of epithelial and neuroendocrine differentiation. The recognition of this cellular phenotype is important for appropriate management of patients.
